# The art of aging well: a study of the relationship between recreational arts engagement, general health and mental wellbeing in cohort of Australian older adults

**DOI:** 10.3389/fpubh.2023.1288760

**Published:** 2023-11-30

**Authors:** Christina R. Davies, Charley A. Budgeon, Kevin Murray, Michael Hunter, Matthew Knuiman

**Affiliations:** ^1^Centre for Arts, Mental Health and Wellbeing WA, School of Allied Health and the School of Humanities, The University of Western Australia, Perth, WA, Australia; ^2^School of Population and Global Health, The University of Western Australia, Perth, WA, Australia; ^3^Busselton Population Medical Research Institute, Busselton, WA, Australia

**Keywords:** arts, general health, mental health, older adult, Warwick-Edinburgh Mental Wellbeing Scale, SF12

## Abstract

**Introduction:**

Evidence of the benefits of arts engagement to community wellbeing has been mounting since the 1990s. However, large scale, quantitative, epidemiological studies of the “arts–healthy aging” relationship, or the types of arts older adults voluntarily choose to engage in as part of their everyday life, for enjoyment, entertainment or as a hobby (vs. therapy or interventions) are limited. The aims of this study were to describe older adult recreational arts engagement via the Busselton Healthy Ageing Study (BHAS) cohort, and to determine if there was an association between arts engagement, general health and mental wellbeing.

**Methods:**

Overall, 2,843 older adults (born 1946–1964) from the BHAS cohort (*n* = 5,107) who had completed a supplementary arts survey (*n* = 3,055, 60%) and had data on required variables were included in this study (93% of those eligible). The dependent variable was general health (SF12) and subjective mental wellbeing (Warwick-Edinburgh Mental Wellbeing Scale, WEMWBS). The independent variable was hours engaged in recreational arts in the last 12 months. A descriptive analysis followed by a linear regression analysis was conducted.

**Results:**

The prevalence of recreational arts engagement in the last 12 months was 85% (mean = 132 h/year). Older adults engaged in the arts in a number of ways including attending events (79%), actively participating/making art (40%), as an arts society/club/organization member (20%), by learning about the arts (13%) or by volunteering/working in the arts (non-professional, 11%). When general health was assessed via the SF12, the average physical component score (PCS) was 50.1 (SD 8.9) and the average mental component score (MCS) was 53.6 (SD 8.3). When mental wellbeing was assessed, the average WEMWBS score was 54.9 (SD = 8.6). After adjustment for 12 demographic and lifestyle covariates, it was found that older adults who engaged in any recreational arts in the last 12 months had significantly higher WEMWBS scores and higher SF12 physical component scores than those who did not engage in the arts (0 h/year).

**Discussion:**

Evidence of an arts-health relationship was found in this study. The suitability of the arts as a population based, healthy aging strategy to influence the mental wellbeing and general health of older adults should be investigated further.

## Introduction

For many people worldwide (1), a long life (60 years and beyond) is now an “expectation” rather than the exception (1, 2). However, as people age into older adulthood they are likely to experience events and risk factors (e.g., reduced income, disability, bereavement, social isolation, loneliness) which have the potential to adversely impact quality of life, health and wellbeing, e.g., loneliness is associated with mental ill-health, high blood pressure and heart disease (3–7). As a result, the 73rd World Health Assembly has called for urgent and concerted global action on healthy aging, especially sustainable solutions that are person-centered and prevention (rather than illness) focused. (3, 4). With an emphasis on self-expression, social inclusion, happiness, agency, enjoyment, and creativity (8–11), like recreational sport (an established health promotion strategy), in Australia, recreational arts engagement is starting to receive attention from government, industry, philanthropists, policy makers, health professionals, artists, and the general community as a public health and healthy aging strategy (10–12).

Recreational arts engagement is an umbrella term that describes the various ways individuals interact with the arts, as part of their everyday life, for enjoyment, entertainment, socially or as a hobby (13, 14). The arts includes a large number of activities/events and has been defined by Davies et al. (13, 15) via five art forms, these being:

- performing arts (e.g., playing a musical instrument, attending a concert, singing, dancing, listening to music);
- visual arts, design and craft (e.g., painting, drawing, pottery, jewelry making, attending an art exhibition);
- community and cultural festivals (i.e., festivals related to community events, national holidays, or events of religious or cultural significance, e.g., Diwali festival, community lantern events, Lunar New Year festivals);
- literature (e.g., storytelling, creative writing, reading novels, book clubs), and
- online, digital and electronic arts (e.g., photography, film making, animation, viewing a movie or e-concert).

Recreational arts engagement flows on a continuum from active involvement (e.g., making art) to receptive involvement (e.g., viewing art) (13, 15). Methods of engagement include (but are not limited to), making, performing, attending, learning about, listening to, viewing art (15) and can occur within a variety of settings, including but not limited to the home, workplaces, schools, community centers, museums, theaters, concert halls, art galleries, parks, places of worship, prisons, aged care facilities, and hospitals (13).

This study responds to the UK all-parliamentary enquiry into the “arts and wellbeing” which found that the arts can help to keep us well and support longer lives better lived (10). This study also responds to the Australian “National Arts and Health Framework” which was endorsed by both Health Ministers and Arts Ministers in every Australian state/territory to promote the integration of the arts into health policy and practice (16), and the recent Australian Royal Commission into aged care that found that many older Australians are isolated, lonely and lack the intellectual activity/resources needed to live a meaningful life (6, 17). Arts interventions and programs for older adults have been found to reduce depressed mood, social isolation, boredom; enhance feelings of joy, happiness, hope, self-worth, relaxation and improve quality of life (14, 18–23). In the general population, qualitative and quantitative studies have found that arts engagement increases perceptions of happiness, resilience, empathy, enjoyment, confidence, self-esteem, self-expression, knowledge, and self-reflection (8, 24–27). Given this, it is now time to assess the effectiveness of arts engagement as a non-pharmacological, healthy aging strategy, and if found to be effective, to utilize the arts in innovative public health policy and practice, like we do with recreational sport (28). While evidence and review of the benefits of arts engagement has been mounting for many years (11, 29–33), large scale, quantitative, epidemiological studies of the “recreational arts–healthy aging” relationship (rather than therapy or as part of an intervention) are limited (8). As a result, this study aimed to (1) describe how older Australian adults engage in recreational arts, and then (2) determine if there is an association between recreational arts engagement (hours per year), general health and mental wellbeing.

## Methods

### Participant recruitment and survey

The study protocol (7) for the Busselton Healthy Ageing Study (BHAS) includes detailed health questionnaires and physical clinical assessments. All non-institutionalized adults (born 1946–1964) listed on the electoral roll as living within the City of Busselton local government electoral boundary were invited to participate (in Australia electoral roll registration is compulsory). Contact was made with 82% of those eligible of which 76% completed the BHAS study protocol at the survey center (*n* = 5,107, 45% male). This cohort provides good power cross-sectionally to study participant health and has similar prevalence of chronic conditions and disease risk factors to the Australian population (7, 34). Based on a previously established survey, arts engagement and mental wellbeing questions were included as a supplementary postal survey in 2015 (8). Permission to conduct this study was granted by the University of Western Australia Human Research Ethics Committee (RA/4/1/2203), and informed consent obtained from participants. Of the 5,107 BHAS cohort participants, 3,055 (60%) replied to the arts survey, of these 2,843 (93%) had the required variables to enable inclusion in this study.

### Dependent variable: general health (SF12) and mental wellbeing (WEMWBS)

#### General health

The SF12 (Version 2, QualityMetric Incorporated) is a multipurpose, short-form measure of health status (35). The SF12 consists of 12 questions that measure eight health domains to assess physical health (PCS) and mental health (MCS). In this study, the PCS and MCS scores were computed and normalized according to published algorithms (35, 36). Scores range from 0 to 100 (mean = 50, SD = 10), with higher scores indicating better physical and mental health. The SF12 has been validated across a number of populations, conditions and has good test–retest reliability (35, 36). Permission to utilize the SF12 was granted under license T122450/OP005134.

#### Mental wellbeing

Subjective mental wellbeing was measured using the 14 item Warwick-Edinburgh Mental Wellbeing Scale (WEMWBS) (37). This scale includes hedonic (e.g., happiness, life satisfaction) and eudaimonic (e.g., positive relationships, psychological functioning) items which together measure mental wellbeing (38). WEMWBS was scored by summing responses to each of the 14 items. Population scores on WEMWBS approximate to a normal distribution, with a minimum score of 14 and a maximum score of 70 (population average = 51) (37). The scale has good validity, test–retest reliability, acceptability and internal consistency (37, 39). Permission to utilize WEMWBS was granted by the University of Warwick.

### Independent variable: hours engaged in recreational arts

**Figure 1:**
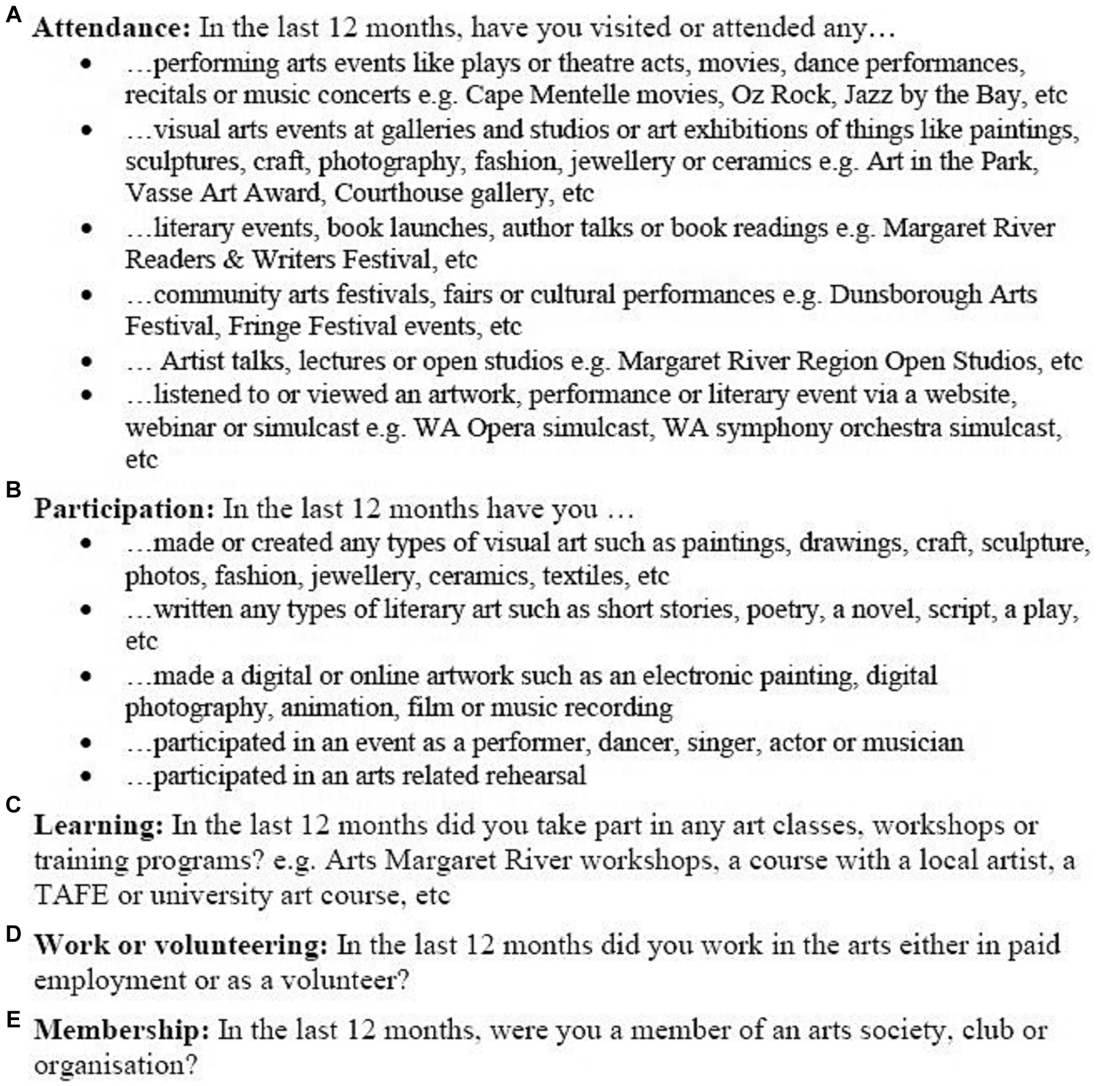
Arts survey questions.

The independent variable in this study was total hours engaged in recreational arts in the last 12 months. Quantifying engagement by asking questions about (1) behavior in the last 12 months, and (2) measurement in terms of “time,” are common in the literature (8). As shown in Figure 1, arts engagement was measured by asking 14 questions about arts attendance (6 questions), arts participation/making (5 questions), learning (1 question), work/volunteering (1 question) and membership (1 question). For each survey item, respondents were asked if they had engaged in the arts in the previous 12 months (yes/no). If “yes,” they were asked to describe the activity or event. Respondents were then asked approximately how many days in the last 12 months they had engaged in each type of arts activity or event, followed by (on a typical day), how many hours they spent engaging.

### Confounding and effect modifying variables

To control for the influence of confounding or effect modification, information about 12 possible covariates to the recreational arts-healthy aging relationship were analyzed (Table 1). This included demographic variables (i.e., sex, age group, household income, education, marital status) and if in the last 12 months respondents experienced a separation/divorce, bereavement, engaged in sport (at least once a week for most weeks), attended religious services/events at a place of worship, partook in a holiday (two or more weeks), experienced financial difficulties or a serious illness.

**Table 1:** BHAS cohort demographic, arts engagement, SF12 and WEMWBS characteristics (*n* = 2843).

Variable	Level	n	%	Arts engagement		SF12				WEMWBS	
						PCS		MCS			
				%	*p*-value	Mean ± SD	*p*-value	Mean ± SD	*p*-value	Mean ± SD	*p*-value
Demographics											
Sex	Male	1215	42.7	77.5	*p* < 0.001	50.3 ± 8.7	NS *p* = 0.392	54.0 ± 8.1	*p* = 0.022	54.5 ± 8.4	*p* = 0.047
	Female	1628	57.3	89.7		50.0 ± 9.1		53.3 ± 8.4		55.2 ± 8.6	
Age group	<50 years	234	8.2	86.3	NS *p* = 0.092	52.9 ± 6.2	*p* < 0.001	52.2 ± 7.9	*p* < 0.001	52.9 ± 9.0	*p* < 0.001
	50 to <55 years	632	22.2	81.3		51.5 ± 7.8		52.4 ± 8.7		53.9 ± 8.3	
	55 to <60 years	725	25.5	83.9		50.9 ± 8.6		53.0 ± 8.5		54.7 ± 8.4	
	60 to <65 years	814	28.6	86.5		48.7 ± 9.6		54.5 ± 8.0		55.7 ± 8.6	
	65 years and over	438	15.4	85.6		47.9 ± 9.9		55.4 ± 7.5		56.5 ± 8.5	
Household income ($AUD)	Refused	322	11.3	87.3*	*p* < 0.001	49.7 ± 9.2*	*p* < 0.001	54.5 ± 8.2*	*p* < 0.001	56.1 ± 8.8*	*p* < 0.001
	<$20,000	164	5.8	79.9		44.3 ± 11.7		51.6 ± 10.5		53.2 ± 9.6	
	$20,001–$40,000	469	16.5	81.9		47.1 ± 10.6		52.6 ± 9.6		53.8 ± 9.2	
	$40,001–$60,000	479	16.8	81.4		50.1 ± 8.5		53.9 ± 8.1		54.8 ± 8.4	
	$60,001–$80,000	369	13	85.6		50.9 ± 7.5		53.8 ± 7.7		54.4 ± 8.1	
	$80,001–$100,000	340	12	85.9		51.7 ± 7.4		53.7 ± 7.1		55 ± 8.2	
	>$100,000	700	24.6	87.0		52.5 ± 7.2		54.0 ± 7.6		55.8 ± 8.1	
Education	High school or less	1325	46.6	77.0	*p* < 0.001	49.4 ± 9.2	*p* < 0.001	53.8 ± 8.6	NS *p* = 0.590	54.6 ± 8.8	*p* < 0.001
	Trade certificate or diploma	896	31.5	88.5		50.0 ± 9.1		53.4 ± 8.2		54.5 ± 8.6	
	University degree or higher degree	622	21.9	94.9		51.8 ± 7.9		53.5 ± 7.7		56.3 ± 7.8	
Married or de-facto relationship	No	449	15.8	84.4	NS *p* = 0.942	49.0 ± 10.0	*p* = 0.004	50.8 ± 10.3	*p* < 0.001	53.4 ± 9.8	*p* < 0.001
	Yes	2394	84.2	84.5		50.3 ± 8.7		54.1 ± 7.7		55.2 ± 8.3	
Recent Divorce or Separation	No	2780	97.8	84.6	NS *p* = 0.252	50.1 ± 8.9	NS *p* = 0.305	53.7 ± 8.1	*p* < 0.001	55.0 ± 8.5	NS *p* = 0.074
	Yes	63	2.2	79.4		51.3 ± 9.2		49 ± 12.1		53.0 ± 10.5	
Recent bereavement	No	2129	74.9	84.0	NS *p* = 0.209	50.2 ± 9	NS *p* = 0.657	54.0 ± 8.1	*p* < 0.001	55.0 ± 8.5	NS *p* = 0.246
	Yes	714	25.1	86.0		50 ± 8.9		52.3 ± 8.8		54.6 ± 8.7	
In the previous 12 months											
Sports engagement	No	595	20.9	70.3	*p* < 0.001	48.1 ± 10	*p* < 0.001	51.6 ± 10.5	*p* < 0.001	51.9 ± 9.6	*p* < 0.001
	Yes	2248	79.1	88.3		50.6 ± 8.6		54.1 ± 7.5		55.7 ± 8.1	
Attended a religious services or event	No	2207	77.6	82.6	*p* < 0.001	50.1 ± 8.9	NS *p* = 0.802	53.4 ± 8.6	*p* = 0.003	54.4 ± 8.6	*p* < 0.001
	Yes	636	22.4	91.2		50.2 ± 9.1		54.5 ± 7.1		56.6 ± 8.2	
Holiday	No	720	25.3	75.1	*p* < 0.001	47.9 ± 10.3	*p* < 0.001	51.7 ± 9.9	*p* < 0.001	52.2 ± 9.4	*p* < 0.001
	Yes	2123	74.7	87.7		50.9 ± 8.3		54.3 ± 7.5		55.8 ± 8.0	
Financial difficulties	No	2576	90.6	84.9	NS *p* = 0.123	50.4 ± 8.6	*p* < 0.001	54.3 ± 7.7	*p* < 0.001	55.3 ± 8.3	*p* < 0.001
	Yes	267	9.4	81.3		47.5 ± 11.5		47.2 ± 10.7		50.9 ± 9.9	
Serious illness	No	2537	89.2	84.5	NS *p* = 0.820	50.8 ± 8.3	*p* < 0.001	53.8 ± 8.0	*p* < 0.001	55.1 ± 8.5	*p* = 0.016
	Yes	306	10.8	85.0		44.3 ± 11.5		52.1 ± 9.8		53.8 ± 9.1	

SD, Standard Deviation; NS, Not statistically significant; PCS, SF12 Physical Composite Score; MCS, SF12 Mental Composite Score. *p*-values for tests involving age, income and education are trend tests, *excluded from trend test.

### Data analysis

The analyses were conducted using SAS^®^ 9.4. Arts “attendance” in the previous 12 months was calculated based on respondent self-reported answers to one or more of the six survey items relating to attendance. Similarly, respondent “participation” was calculated based on responses to one or more of the five survey items related to participation. A respondent was considered to be engaged in the arts in the previous 12 months (prevalence) if they had attended, and/or participated, and/or took part in arts learning, and/or worked/volunteered in the arts (non-professional basis) and/or been a member of an arts organization, club or society. “Total days engaged in the arts in the previous 12 months” was calculated by summing the number of days respondents spent attending, participating, learning, working/volunteering or being a member. “Hours per *day* engaged in the arts in the previous 12 months,” was calculated by first multiplying hours on a typical day by number of days engaged in each arts activity over the last 12 months, this was then summed and the total divided by the sum of days engaged in each arts activity. “Hours per *year* engaged in the arts,” was calculated by multiplying hours on a typical day by number of days engaged in each activity in the previous 12 months and summing each sub-total.

Summary statistics were calculated for categorical variables and means and standard deviations for continuous variables. Chi-squared tests and trend tests were carried out to initially investigate the potential correlates of arts engagement. Linear regression was then used to investigate associations between arts engagement (hours per year), SF12 (PCS, MCS) and WEMWBS. As the distribution of arts engagement was positively skewed (i.e., 16% did not engage in the arts, median = 23 h/year, 75th percentile = 102 h/year), “hours per *year* engaged in the arts” was grouped into four categories: no art = 0 h/year, low arts engagement = 0.1 to 22.9 h/year, medium arts engagement = 23 to 101.9 h/year and high arts engagement = 102 or more hours/year (8). Overall, three models were fitted. The first model estimated the direct (unadjusted) effect of arts engagement (Model A). The second model (Model B) estimated the effect of arts engagement after adjustment for demographics. The third model (Model C) adjusted for demographics, plus separation/divorce, bereavement, sports engagement, religious activities, holidays, financial difficulties, and serious illness. Interactions between sex and arts engagement were also examined.

## Results

Table 1 shows cohort characteristics by demographics, arts engagement, SF12, and WEMWBS (*n* = 2,843). Overall, 57% of subjects were female. Most were married or in a *de-facto* relationship (84%), had not recently separated/divorced (98%), not suffered a recent bereavement (75%), not experienced financial difficulties (91%), and had not experienced a serious illness (89%). Approximately half of all subjects completed more than a high school education (53%) and half had an annual income greater than AUD$60,000 per year (50%). Most subjects engaged in sport (79%), had taken a holiday (75%), and had not attended a religious service/event (78%) in the last 12 months.

### Recreational arts engagement

As shown in Table 2, in the previous 12 months, 79% of subjects had attended an arts event; 40% actively participated/made art; 13% took part in arts related learning; 11% worked or volunteered in the arts (non-professional basis) and 20% were an arts society/club member. In the previous 12 months, on average, respondents spent 17 h attending arts events; 57 h participating/making art; 10 h learning about the arts; 19 h working/volunteering in the arts (non-professional) and 30 h as an arts society/club member. Overall, the prevalence of recreational arts engagement in the cohort was 85%. The cohort spent an average of 132 h per year (SD 330, median 23) engaging in recreational arts activities or events. As the distribution of arts engagement hours was skewed it was grouped. Overall, 16% of respondents did not engage in the arts, 34% had low arts engagement (0.1–22.99 h/year), 25% had medium arts engagement (23–101.99 h/year) and 25% had high arts engagement (102 or more hours/year) (8). As shown in Table 1, females were significantly more likely to engage in recreational arts than males (*p* < 0.001). Recreational arts engagement also significantly increased by income (*p* < 0.001), education (*p* < 0.001), and was more likely in those who also engaged in sport (*p* < 0.001), who attended religious services/events (*p* < 0.001) and had taken a holiday (*p* < 0.001) in the previous 12 months.

**Table 2:** BHAS cohort prevalence, days and hours engaged in the arts in the previous 12 months (*n* = 2,843).

Arts engagement measure		Attendance	Participation/Making	Learning	Work or volunteer*	Member	Overall arts engagement
Prevalence of arts engagement in the previous 12 months		78.9%	39.5%	13.4%	10.7%	20.1%	84.5%
Days engaged in the arts in the previous 12 months	Mean	6.4	17.8	2.8	4.2	8.5	39.4
	SD	13.4	49.01	16.5	24.9	28.7	77.9
Hours per day, engaged in the arts, in the previous 12 months	Mean	2.0	1.2	0.6	0.4	0.7	4.8
	SD	1.5	1.8	1.7	1.4	1.6	4.8
Hours engaged in the arts in the previous 12 months	Mean	17.0	57.5	10.4	19.2	29.9	132.2
	SD	46.9	191.8	61.9	141.9	121.2	330.2

*Non-professional art engagement (i.e., recreational, not a professional artist), SD, standard deviation.

### General health and recreational arts engagement (SF12)

The average SF12 physical component score (PCS) for respondents was 50.1 (SD 8.9) and the average mental component score (MCS) was 53.6 (SD 8.3). As shown in Table 1, PCS significantly decreased by age group (*p* < 0.001) and increased by income (*p* < 0.001), education (*p* < 0.001), and marital status (*p* = 0.004). MCS were higher in males (*p* = 0.02) and significantly increased by age group (*p* < 0.001), income (*p* < 0.001), marital status (*p* < 0.001) and not recently experiencing a divorce/separation (*p* < 0.001) or bereavement (*p* < 0.001). PCS and MCS were higher in those who engaged in sport (*p* < 0.001), had taken a holiday (*n* < 0.001), had no financial difficulties (*p* < 0.001) or serious illness (*p* < 0.001) in the previous 12 months. MCS was also higher in those who had attended a religious service/event in the last 12 months (*p* = 0.003).

As shown in Table 3, Model A (unadjusted), respondents who did not engage in the arts in the last 12 months (PCS 48.4, MCS 52.6), on average, had lower physical and mental component scores than those who had low (PCS 50.8, MCS 54.0), medium (PCS 50.8, MCS 53.6) or high (PCS 49.6, MCS 53.7) levels of recreational arts engagement. After adjustment for demographics, separation or divorce, bereavement, sports engagement, religious activities, holidays, financial difficulties, serious illness (Overall Test–2 groups, Model C) those who did not engage in the arts in the last 12 months (0 h/year) had significantly lower PCS than those who engaged in “any” recreational arts (mean difference − 1.1, 95% CI [−2.0, −0.2], *p* = 0.016) however a statistically significant difference in MCS was not found in the fully adjusted model (*p* = 0.337). There was no clear trend in PCS or MCS (unadjusted or adjusted) means with increasing category of arts engagement, with the “low” category of arts engagement having the highest means. Effect modification by sex was not found to be significant.

**Table 3:** Unadjusted and adjusted associations between recreational arts engagement (hrs/year), general health (SF12 PCS, MCS) and mental wellbeing (WEMWBS) (*n* = 2,843).

	Regression models																	
Measure	PCS						MCS						WEMWBS					
	Model A—unadjusted model		Model B—adjusted for demographics		Model C—adjusted for demographics, and events in the last 12 months		Model A—unadjusted model		Model B—adjusted for demographics		Model C—adjusted for demographics, and events in the last 12 months		Model A—unadjusted model		Model B—adjusted for demographics		Model C—adjusted for demographics, and events in the last 12 months	
Arts engagement*	Mean (95% CI)	*p*-value	Mean (95% CI)	*p*-value	Mean (95% CI)	*p*-value	Mean (95% CI)	*p*-value	Mean (95% CI)	*p*-value	Mean (95% CI)	*p*-value	Mean (95% CI)	*p*-value	Mean (95% CI)	*p*-value	Mean (95% CI)	*p*-value
None (*n* = 440)	48.4 (47.6, 49.3)	<0.001	48.7 (47.8, 49.7)	0.002	46.7 (45.2, 48.1)	0.030	52.6 (51.8, 53.4)	0.032	51.3 (50.4, 52.2)	0.054	47.9 (46.6, 49.2)	0.502	51.2 (50.4, 52.0)	<0.001	50.6 (49.7, 51.5)	<0.001	49.4 (48.0, 50.7)	<0.001
Low (*n* = 973)	50.8 (50.2, 51.3)		50.6 (49.9, 51.3)		48.0 (46.7, 49.3)		54.0 (53.5, 54.5)		52.6 (51.9, 53.2)		48.5 (47.2, 49.7)		54.9 (54.4, 55.4)		53.9 (53.3, 54.6)		51.7 (50.5, 53.0)	
Medium (*n* = 716)	50.8 (50.2, 51.4)		50.4 (49.7, 51.2)		47.7 (46.4, 49.0)		53.6 (53.0, 54.2)		52.2 (51.5, 52.9)		48.0 (46.7, 49.3)		55.8 (55.2, 56.5)		54.8 (54.0, 55.5)		52.4 (51.1, 53.7)	
High (*n* = 714)	49.6 (48.9, 50.2)		50.0 (49.2, 50.8)		47.3 (45.9, 48.6)		53.8 (53.2, 54.4)		52.5 (51.7, 53.2)		48.3 (47.1, 49.6)		56.3 (55.7, 56.9)		55.2 (54.5, 56.0)		52.8 (51.5, 54.1)	

Pairwise comparisons^	Mean difference (95% CI)	*p*-value	Mean difference (95% CI)	*p*-value	Mean difference (95% CI)	*p*-value	Mean difference (95% CI)	*p*-value	Mean difference (95% CI)	*p*-value	Mean difference (95% CI)	*p*-value	Mean difference (95% CI)	*p*-value	Mean difference (95% CI)	*p*-value	Mean difference (95% CI)	*p*-value
None vs. Low	-2.3 (-3.3, -1.3)	<0.001	-1.9 (-2.9, -0.9)	<0.001	-1.4 (-2.4, -0.4)	0.005	-1.4 (-2.3, -0.5)	0.004	-1.3 (-2.2, -0.3)	0.008	-0.6 (-1.5, 0.3)	0.219	-3.7 (-4.6, -2.8)	<0.001	-3.3 (-4.3, -2.4)	<0.001	-2.4 (-3.3, -1.4)	<0.001
None vs. Medium	-2.4 (-3.4, -1.3)	<0.001	-1.7 (-2.8, -0.6)	0.002	-1.1 (-2.1, 0.0)	0.051	-1.0 (-1.9, 0.0)	0.053	-0.9 (-1.9, 0.1)	0.081	-0.1 (-1.1, 0.9)	0.823	-4.6 (-5.6, -3.6)	<0.001	-4.1 (-5.2, -3.1)	<0.001	-3.1 (-4.1, -2.0)	<0.001
None vs. High	-1.1 (-2.2, -0.1)	0.034	-1.3 (-2.3, -0.2)	0.022	-0.6 (-1.7, 0.4)	0.244	-1.2 (-2.2, -0.2)	0.019	-1.2 (-2.2, -0.2)	0.022	-0.4 (-1.4, 0.6)	0.392	-5.1 (-6.1, -4.1)	<0.001	-4.6 (-5.7, -3.6)	<0.001	-3.5 (-4.5, -2.4)	<0.001
Low vs. Medium	0 (-0.9, 0.8)	0.933	0.2 (-0.6, 1.0)	0.632	0.3 (-0.5, 1.1)	0.416	0.4 (-0.4, 1.2)	0.310	0.4 (-0.4, 1.2)	0.360	0.5 (-0.3, 1.2)	0.237	-0.9 (-1.8, -0.1)	0.023	-0.8 (-1.6, -0.0)	0.044	-0.7 (-1.5, 0.1)	0.092
Low vs. High	1.2 (0.3, 2.0)	0.007	0.7 (-0.2, 1.5)	0.131	0.7 (-0.1, 1.6)	0.075	0.2 (-0.6, 1.0)	0.613	0.1 (-0.7, 0.9)	0.842	0.1 (-0.6, 0.9)	0.732	-1.4 (-2.2, -0.6)	0.001	-1.3 (-2.1, -0.5)	0.002	-1.1 (-1.9, -0.3)	0.007
Medium vs. High	1.2 (0.3, 2.1)	0.009	0.4 (-0.4, 1.3)	0.327	0.4 (-0.5, 1.3)	0.354	-0.2 (-1.1, 0.7)	0.637	-0.3 (-1.1, 0.6)	0.507	-0.3 (-1.1, 0.5)	0.436	-0.5 (-1.3, 0.4)	0.295	-0.5 (-1.3, 0.4)	0.272	-0.4 (-1.3, 0.4)	0.324

**Overall Test—2 groups** “None” vs. “Any” recreational arts engagement^#^																		
Arts engagement*																		
None (No. art)	48.4 (47.6, 49.3)	<0.001	48.7 (47.8, 49.6)	<0.001	46.6 (45.2, 48.0)	0.016	52.6 (51.8, 53.4)	0.005	51.3 (50.4, 52.2)	0.009	47.9 (46.5, 49.2)	0.337	51.2 (50.4, 52.0)	<0.001	50.7 (49.8, 51.6)	<0.001	49.5 (48.1, 50.8)	<0.001
Any art^#^	50.4 (50.1, 50.8)		50.4 (49.8, 50.9)		47.7 (46.5, 49.0)		53.8 (53.5, 54.1)		52.4 (51.9, 52.9)		48.3 (47.1, 49.4)		55.6 (55.3, 55.9)		54.6 (54.0, 55.1)		52.3 (51.1, 53.5)	
Pairwise comparisons^																		
Non vs. Any art^#^	-2.0 (-2.9, -1.1)	<0.001	-1.7 (-2.6, -0.8)	<0.001	-1.1 (-2.0, -0.2)	0.016	-1.2 (-2.0, -0.4)	0.005	-1.1 (-2.0, -0.3)	0.009	-0.4 (-1.3, 0.4)	0.337	-4.4 (-5.3, -3.5)	<0.001	-3.9 (-4.8, -3.0)	<0.001	-2.8 (-3.7, -1.9)	<0.001

*Adjusted means (95% confidence intervals) displayed. ^Mean differences (95% confidence intervals) displayed. ^#^Any recreational arts =low + medium + high levels of arts engagement. Model A—unadjusted effect of arts engagement on general health and mental wellbeing. Model B—effect of arts engagement on general health and mental wellbeing after adjustment for specific demographics (i.e., sex, age group, household income, education, marital status). Model C—effect of arts engagement on general health and mental wellbeing after adjustment for demographics (i.e., sex, age group, household income, education, marital status) and events in the last 12 months (i.e., separation/divorce, bereavement, sports engagement, religious activities, holidays, financial difficulties and serious illness).

### Mental wellbeing and recreational arts engagement (WEMWBS)

The WEMWBS mean score for the cohort was 54.9 (SD 8.6). As shown in Table 1, females had a higher average WEMWBS scores than males (*p* = 0.047), as did older compared to younger subjects (*p* < 0.001). Average WEMWBS scores also significantly increased by income (*p* < 0.001), education (*p* < 0.001) and marital status (*p* < 0.001) and were higher for those who engaged in sport (*p* < 0.001), attended a religious service/event (*p* < 0.001), had taken a holiday (*n* < 0.001), who had no financial difficulties (*p* < 0.001) or serious illness (*p* = 0.016) in the previous 12 months.

As shown in Table 3, Model A (unadjusted), older adults who engaged in low (54.9), medium (55.8) or high (56.3) levels of arts engagement in the last 12 months had significantly higher WEMWBS scores than those who did not engage in the arts (51.2). After adjustment for demographics, separation or divorce, bereavement, sports engagement, religious activities, holidays, financial difficulties, serious illness (Overall Test–2 groups, Model C), it was found that people who did not engage in recreational arts (0 h/year) had significantly lower WEMWBS scores than those who engaged in “any” recreational arts in the last 12 months (mean difference − 2.8, 95% CI [−3.7, −1.9], *p* < 0.001). In general, respondents who participated in any (low, medium or high) recreational arts had WEMWBS scores almost three points higher than those who did not engage in recreational arts. Further, there was evidence in both unadjusted and adjusted means of increasing WEMWBS scores with higher levels of arts engagement with the “high” category of arts engagement having the highest means. Effect modification by sex was not found to be significant.

## Discussion

This study contributes to the healthy aging literature and increases knowledge of the impact of recreational arts engagement on older adult mental wellbeing and general health. Encouraging older adults to “shift” their behavior to encourage actions that enhance wellbeing is a common health promotion strategy as this approach, at a population level, can benefit more older adults (overall) than targeting only specific groups. For example, a population approach to increasing physical health might be to encourage older adults to engage in adequate levels of exercise such as walking, balance and strength-based exercise. Likewise, population-based strategies to improve older adult mental wellbeing and general health could be to encourage recreational arts-based activities, programs and events in the community and in aged care, e.g., creative writing, book clubs, painting, pottery, knitting, coloring, photography, playing a musical instrument, singing, dancing, art classes, attending concerts, etc.

In this study, a significant association between arts engagement and mental wellbeing, as measured by WEMWBS, was found. This is consistent with the arts-mental health literature for both the general population and interventions with older adults (8, 40, 41). After adjustment for demographic and lifestyle factors, respondents who engaged in any recreational arts in the last 12 months had significantly higher WEMWBS scores than those who did not engage at all. Evidence of increasing WEMWBS scores with higher levels of arts engagement was found. Respondents with “high” arts engagement in the last 12 months (102 h or more/year, or 2 h or more/week) had average WEMWBS score 3.5 points higher than those who did not engage at all (0 h/year). WEMWBS responsiveness to change has been assessed at both the individual and population level for which a difference of three or more points can be considered meaningful (39).

When the association between arts engagement in the last 12 months, and general health was assessed, a weaker relationship was found for the SF12 physical component, but not for the mental health component. The impact of arts engagement on general health, as measured by the SF12, has had mixed findings in the literature. For example, some arts engagement studies have showed a significant increase in SF12 mental health component scores at 6-month follow-up (18, 42) but not at 12-month follow-up (18), and for SF12 physical component scores, no significant change at 6-month follow-up (18, 42) but “close to significant” (*p* = 0.06) change at 12-month follow-up (18). One explanation for our results and the literature, is that arts related physical health changes may take longer than 6 months to develop. Acknowledging that (1) our results are an association and not an indication of causality or relationship directionality (i.e., older adult arts engagement may improve mental wellbeing and/or older mental wellbeing may increase the likelihood of engagement in the arts), and (2) given the decline in our physical health as we age (3–7), rigorous research (e.g., RCTs, prospective cohort studies) is needed to assess whether recreational arts-based activities, programs and events can positively impact the physical health of older adults living in the community and in aged care. One possible explanation for our SF12 mental health component result and the literature, is that the mental health effects of arts engagement are short term, however, this is not consistent with our results for WEMWBS. Keeping in mind that when asked to self-report outcomes obtained from engaging in the arts, members of the general population are more likely to mention positive (e.g., happiness, relaxation, self-esteem) rather than negative outcomes (e.g., anxiety, frustration, disappointment) (25). Another explanation for the difference in results between WEMWBS and the SF12 mental health component, is because these surveys are measuring different mental health outcomes. The Warwick-Edinburgh Mental Wellbeing Scale was developed to measure positive mental health (39), that is, WEMWBS measures positive mental health or mental wellbeing, via hedonic (e.g., happiness) and eudaimonic questions (e.g., positive relationships) (37, 38). The mental health component of the SF12 however measures mental health functioning and low scores can be used as an indicator of ill-health (35). More research is needed to determine which mental health measures are most appropriate when conducting studies related to arts engagement.

Overall, this paper provides three important findings for government, industry, philanthropists, policy makers, health professionals, artists, older adults, with results generalizable to Australian states/territories and countries with similar mental-health, health and socio-demographic profiles to Australia. First, recreational arts engagement may have the potential to enhance the mental wellbeing and general health of older adults. Second, when engaging in creative activities and events, more research is needed to determine the “art dose” or the optimal amount of time older adults should spend engaging in the arts, e.g., in the general population (18 years and older) it has been suggested that two or more hours/week of arts engagement is associated with enhanced mental wellbeing (8). Third, in time, if the relationship between hours engaged in the arts, general health and mental wellbeing is found to be causal, there is potential for innovative, non-pharmacological, “arts-healthy aging” policy, programs, campaigns and strategies, such as those used to promote the health benefits of sport and physical activity to older Australians. For example, in Western Australia, Injury Matters and the Department of Health have implemented the “Stay on your Feet”^®^ falls prevention campaign to promote the health benefits of physical activity to health professionals, older adults, and the friends and families of older adults (43).

## Conclusion

Strengths of this study include its large sample size, high response, adjustment for a wide range of confounders and effect modifiers to the arts-healthy aging relationship and that the cohort is representative of the Australian population. A limitation of this study is that our analyses are cross-sectional and therefore preclude our ability to determine causality. For researchers to determine whether or not a causal relationship exists between arts engagement and healthy aging study designs that consider temporal order are needed (e.g., RCTs) (44). Enablers and barriers to the arts-healthy aging relationship should also be investigated (e.g., cost, access, someone to attend arts events with), as should the influence of arts engagement methods (i.e., active vs. receptive) to determine which elements have the most impact on mental wellbeing and general health.

## Data availability statement

The datasets presented in this article are not readily available. The Busselton Population Medical Research Institute (BPMRI) welcomes applications from researchers for data access which are considered by the BPMRI Scientific Research Committee.

## Ethics statement

The study was approved by the University of Western Australia Human Research Ethics Committee (RA/4/1/2203). The studies were conducted in accordance with the local legislation and institutional requirements. The participants provided their written informed consent to participate in this study.

## Author contributions

CD: Conceptualization, Data curation, Formal analysis, Funding acquisition, Investigation, Methodology, Project administration, Resources, Software, Supervision, Validation, Visualization, Writing – original draft, Writing – review & editing. CB: Data curation, Formal analysis, Investigation, Project administration, Software, Writing – original draft, Writing – review & editing. KM: Data curation, Formal analysis, Funding acquisition, Investigation, Project administration, Software, Writing – original draft, Writing – review & editing. MH: Data curation, Investigation, Methodology, Resources, Software, Validation, Writing – original draft, Writing – review & editing. MK: Conceptualization, Data curation, Formal analysis, Funding acquisition, Investigation, Methodology, Project administration, Resources, Software, Validation, Writing – original draft, Writing – review & editing.

## References

[1] World Health Organization. Ageing and health. Geneva: WHO (2021) Available at: https://www.who.int/news-room/fact-sheets/detail/ageing-and-health.

[2] Higgs P Gilleard C. Frailty, abjection and the ‘othering’ of the fourth age. Health Sociol Rev. (2014) 23:10–9. doi: 10.5172/hesr.2014.23.1.10, PMID: 27912849

[3] United Nations- Department of Economic and Social Affairs. World population aging 2019. New York, NY: United Nations. (2020). Available at: https://www.un.org/en/development/desa/population/publications/pdf/ageing/WorldPopulationAgeing2019-Report.pdf (Accessed October, 2020).

[4] World Health Organisation. Decade of healthy ageing (2020-2030) (2020). Available at: https://www.who.int/ageing/decade-of-healthy-ageing.

[5] National Institute on Aging. Social isolation, loneliness in older people pose health risks (2019). Available at: https://www.nia.nih.gov/news/social-isolation-loneliness-older-people-pose-health-risks.

[6] Commonwealth of Australia. Royal commission into aged care quality and safety - interim report volume 1 Australian Government (2019) Available at: https://agedcare.royalcommission.gov.au/publications/Documents/interim-report/interim-report-volume-1.pdf.

[7] The Busselton Healthy Ageing Study (BHAS) Investigator Group James A Hunter M Straker L Beilby J Bucks R. Rationale, design and methods for a community-based study of clustering and cumulative effects of chronic disease processes and their effects on ageing: the Busselton healthy ageing study. BMC Public Health. (2013) 13:1–12. doi: 10.1186/1471-2458-13-936, 24099269 PMC3852572

[8] Davies C Knuiman M Rosenberg M. The art of being mentally healthy: a study to quantify the relationship between recreational Arts engagement and mental well-being in the general population. BMC Public Health. (2016) 16:15. doi: 10.1186/s12889-015-2672-7 26733272 PMC4702355

[9] Smith R. Spend (slightly) less on health and more on the arts. BMJ. (2002) 325:1432–3. doi: 10.1136/bmj.325.7378.1432, PMID: 12493650 PMC1124894

[10] All-Party Parliamentary Group on Arts Health and Wellbeing. Creative health: the arts for health and wellbeing (short report) (2017). Available at: http://www.artshealthandwellbeing.org.uk/appg-inquiry/.

[11] Davies C Pescud M. The Arts, creative industries and health: an evidence check rapid review brokered by the sax Institute for the Victorian Health Promotion Foundation. New South Wales: Sax Institute (2020).

[12] Commonwealth of Australia. Revive: a place for every story, a story for every place – Australia’s cultural policy for the next five years. Canberra, ACT: Australian Government (2023) Available at: https://www.arts.gov.au/sites/default/files/documents/national-culturalpolicy-8february2023.pdf.

[13] Davies C Clift S. Arts and Health Glossary - a summary of definitions for use in research, policy and practice. Front Psychol. (2022) 13:949685. doi: 10.3389/fpsyg.2022.949685, PMID: 35936315 PMC9354682

[14] Archibald M Kitson A. Using the arts for awareness, communication and knowledge translation in older adulthood: a scoping review. Arts Health. (2019) 3:1–17. doi: 10.1080/17533015.2019.1608567 31046603

[15] Davies C Rosenberg M Knuiman M Ferguson R Pikora T Slatter N. Defining arts engagement for population-based health research: art forms, activities and level of engagement. Arts Health. (2012) 4:203–16. doi: 10.1080/17533015.2012.656201

[16] Standing Council on Health and Cultural Ministers. National arts and health framework (2014) Available at: http://mcm.arts.gov.au/national-arts-and-health-framework.

[17] Pagone T Care BL. Dignity and respect: the final report of the royal commission into aged care quality and safety. Canberra, ACT: Commonwealth of Australia (2021) Available at: https://agedcare.royalcommission.gov.au/publications/final-report.

[18] Greaves C Farbus L. Effects of creative and social activity on the health and well-being of socially isolated older people: outcomes from a multi-method observational study. J R Soc Promot Heal. (2006) 126:134–42. doi: 10.1177/1466424006064303, PMID: 16739619

[19] MacLeod A Skinner M Wilkinson F Reid H. Connecting socially isolated older rural adults with older volunteers through expressive arts. Can J Aging. (2016) 35:14–27. doi: 10.1017/S071498081500063X, PMID: 26934547

[20] Anderson S Fast J Keating N Eales J Chivers S Barnet D. Translating knowledge: promoting health through intergenerational community Arts programming. Health Promot Pract. (2017) 18:15–25. doi: 10.1177/1524839915625037, PMID: 26933005

[21] Petrovsky DV Sefcik JS Cacchione PZ. A qualitative exploration of choral singing in community-dwelling older adults. West J Nurs Res. (2020) 42:340–7. doi: 10.1177/0193945919861380, PMID: 31256749 PMC6935434

[22] Fraser K O'Rourke H Wiens H Lai J Howell C Brett-MacLean P. A scoping review of research on the Arts, aging, and quality of life. Gerontologist. (2015) 55:719–29. doi: 10.1093/geront/gnv027, PMID: 26179707

[23] Edwards L Owen-Booth B. An exploration of engagement in community based creative activities as an occupation for older adults. Irish J Occup Ther. (2021) 49:51–7. doi: 10.1108/IJOT-05-2020-0009, PMID: 33887950

[24] Australia Council for the Arts. Connecting Australians: results of the national arts participation survey (2017). Available at: https://www.australiacouncil.gov.au/workspace/uploads/files/connecting-australians-natio-59520692c614a.pdf

[25] Davies C Knuiman M Wright P Rosenberg M. The art of being healthy: a qualitative study to develop a thematic framework for understanding the relationship between health and the arts. BMJ Open. (2014) 4:e004790–10. doi: 10.1136/bmjopen-2014-004790, PMID: 24770587 PMC4010846

[26] A New Approach (ANA). Transformative: impacts of culture and creativity acton ACT: produced by ANA think tank with lead delivery partner the Australian Academy of the Humanities. (2019). Available at: https://www.humanities.org.au/new-approach/report2/.

[27] Boydell KM Croguennec J. A creative approach to knowledge translation: the use of short animated film to share stories of refugees and mental health. Int J Environ Res Public Health. (2022) 19. doi: 10.3390/ijerph191811468, PMID: 36141741 PMC9517506

[28] McPhee J French D Jackson D Nazroo J Pendleton N Degens H. Physical activity in older age: perspectives for healthy ageing and frailty. Biogerontology. (2016) 17:567–80. doi: 10.1007/s10522-016-9641-0, PMID: 26936444 PMC4889622

[29] White M. Arts development in community health: a social tonic. Oxon: Radcliffe Publishing (2009). 10.7748/ns2010.04.24.33.30.b1046 27145246

[30] Clift S Camic P eds. Oxford textbook of creative Arts, health, and wellbeing: international perspectives on practice, policy and research. Oxford: Oxford University Press (2016).

[31] Zarobe L Bungay H. The role of arts activities in developing resilience and mental wellbeing in children and young people a rapid review of the literature. Perspect Public Health. (2017) 137:337–47. doi: 10.1177/1757913917712283, PMID: 28613107

[32] Fancourt D Finn S. What is the evidence on the role of the arts in improving health and well-being? A scoping review, health evidence network synthesis report 67. Copenhagen: WHO Regional Office for Europe (2019). 32091683

[33] Zbranca R Dâmaso M Blaga O Kiss K Dascl M Yakobson D. CultureForHealth report: culture’s contribution to health and well-being: a report on evidence and policy recommendations for Europe. Brussels: Culture Action Europe (2022). Available at: https://www.cultureforhealth.eu/app/uploads/2023/02/Final_C4H_FullReport_small.pdf.

[34] Hunter M Knuiman M Musk B Hui J Murray K Beilby J. Prevalence and patterns of multimorbidity in Australian baby boomers: the Busselton healthy ageing study. BMC Public Health. (2021) 21:1539. doi: 10.1186/s12889-021-11578-y, PMID: 34380465 PMC8359115

[35] Ware J Kosinski M Keller S. SF-12: how to score the SF-12 physical and mental summary scales. 2nd ed. Boston, MA: The Health Institute, New England Medical Center (1995).

[36] Ware J Kosinski M Keller S. A 12-item short-form health survey: construction of scales and preliminary tests of reliability and validity. Med Care. (1996) 34:220–33. doi: 10.1097/00005650-199603000-00003, PMID: 8628042

[37] Stewart-Brown S Janmohamed K. Warwick-Edinburgh Mental Well-being Scale (WEMWBS) user guide: Version 1 (2008). Available at: http://www.cppconsortium.nhs.uk/admin/files/1343987601WEMWBS%20User%20Guide%20Version%201%20June%202008.pdf.

[38] Clarke A Friede T Putz R Ashdown J Martin S Blake A. Warwick-Edinburgh Mental Well-Being Scale (WEMWBS): validated for teenage school students in England and Scotland. A mixed methods assessment. BMC Public Health. (2011) 11:487. doi: 10.1186/1471-2458-11-487, PMID: 21693055 PMC3141456

[39] Maheswaran H Weich S Powell J Stewart-Brown S. Evaluating the responsiveness of the Warwick Edinburgh Mental Well-Being Scale (WEMWBS): group and individual level analysis. Health Qual Life Outcomes. (2012) 10:156. doi: 10.1186/1477-7525-10-156, PMID: 23270465 PMC3560098

[40] Beauchet O Cooper-Brown L Hayashi Y Deveault M Launay C. Improving the mental and physical health of older community-dwellers with a museum participatory art-based activity: results of a multicentre randomized controlled trial. Aging Clin Exp Res. (2022) 34:1645–54. doi: 10.1007/s40520-022-02139-3, PMID: 35578103

[41] Beauchet O Bastien T Mittelman M Hayashi Y Ho AHY. Participatory art-based activity, community-dwelling older adults and changes in health condition: results from a pre-post intervention, single-arm, prospective and longitudinal study. Maturitas. (2020) 134:8–14. doi: 10.1016/j.maturitas.2020.01.006, PMID: 32143777

[42] Coulton S Clift S Skingley A Rodriguez J. Effectiveness and cost-effectiveness of community singing on mental health-related quality of life of older people: randomised controlled trial. Br J Psychiatry. (2015) 207:250–5. doi: 10.1192/bjp.bp.113.129908, PMID: 26089304

[43] Injury Matters I. Stay on your feet: move, improve, remove. Leederville, WA: IM (2023). Available at: https://www.injurymatters.org.au/programs/stay-on-your-feet/.

[44] Bradford HA. The environment and disease: association or causation? Proc R Soc Med. (1965) 58:295–300. doi: 10.1177/003591576505800503 14283879 PMC1898525

